# Probing neural circuit mechanisms in Alzheimer’s disease using novel technologies

**DOI:** 10.1038/s41380-023-02018-x

**Published:** 2023-03-23

**Authors:** Steven F. Grieco, Todd C. Holmes, Xiangmin Xu

**Affiliations:** 1 Department of Anatomy and Neurobiology, School of Medicine, University of California, Irvine, CA 92697 USA; 2 Center for Neural Circuit Mapping (CNCM), University of California, Irvine, CA 92697 USA; 3 Department of Physiology and Biophysics, School of Medicine, University of California, Irvine, CA 92697 USA

**Keywords:** Neuroscience, Genetics

## Abstract

The study of Alzheimer’s Disease (AD) has traditionally focused on neuropathological mechanisms that has guided therapies that attenuate neuropathological features. A new direction is emerging in AD research that focuses on the progressive loss of cognitive function due to disrupted neural circuit mechanisms. Evidence from humans and animal models of AD show that dysregulated circuits initiate a cascade of pathological events that culminate in functional loss of learning, memory, and other aspects of cognition. Recent progress in single-cell, spatial, and circuit omics informs this circuit-focused approach by determining the identities, locations, and circuitry of the specific cells affected by AD. Recently developed neuroscience tools allow for precise access to cell type-specific circuitry so that their functional roles in AD-related cognitive deficits and disease progression can be tested. An integrated systems-level understanding of AD-associated neural circuit mechanisms requires new multimodal and multi-scale interrogations that longitudinally measure and/or manipulate the ensemble properties of specific molecularly-defined neuron populations first susceptible to AD. These newly developed technological and conceptual advances present new opportunities for studying and treating circuits vulnerable in AD and represent the beginning of a new era for circuit-based AD research.

## Introduction

Alzheimer’s disease (AD) is the most common cause of memory decline in the elderly and affects ~50 million people worldwide [1]. The management of AD is of major socioeconomic concern as the elderly population is projected to double by 2060 [2], and so the World Health Organization (WHO) has made AD a major priority [3]. Billions of dollars of R&D spending have led to over 100 drug development ventures with equivocal outcomes [4, 5]. In 2021 the FDA approved aducanumab (Aduhelm; Biogen, Inc.) as the first new drug for AD in two decades. Although aducanumab garnered much interest, its efficacy and cost/benefit ratio is not well established and is thus controversial [6]. Drug development failures show that we need new approaches and conceptual frameworks [7, 8]. Recently, clinicians and basic researchers have identified neural circuit dysregulation as an early feature of AD, before other pathological features are measurable [9, 10]. This new evidence provokes the question “Is AD a circuit disease?” [11], and suggests a new conceptual framework for AD research.

In this forward-thinking review article, we provide a synthesis of newly emerging technologies and concepts. This synthesis encourages neuroscientists to address how AD may be a disease of neural circuits [12]. We review single-cell, spatial, and circuit omics approaches for characterizing the identities, locations, and circuits of the cells affected by AD. Next, we describe the use of neural circuit manipulation techniques to functionally test cell type-specific circuit contributions to AD. We then show how a more complete understanding of the circuits impacted by AD is being developed using multimodal and multi-scale approaches. Finally, we discuss progress in novel therapy for circuit disorders. We end our discussion by summarizing conclusions, outstanding questions and future directions related to the circuit-basis of AD.

### The Entorhinal-Hippocampal system

**Fig. 1:**
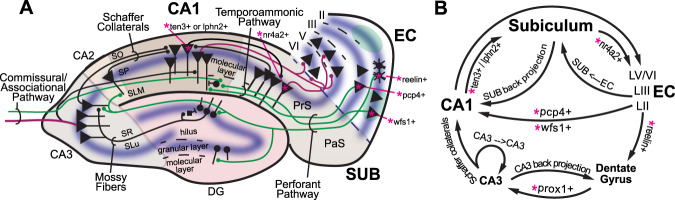
Entorhinal-hippocampal cell type-specific circuits. **A** Schematic illustration of the specific cell types participating in entorhinal-hippocampal circuitry mapped onto the anatomic organization (please see details in Valero and de la Prida 2018). Green axons represent inputs to hippocampus and red axons are outputs. Recently, it has been discovered that CA1 cells that express teneurin-3 (Ten3+) or latrophilin-2 (Lphn2+) project to subiculum (SUB)(please see Berns et al., 2018; and Pederick et al. 2021). From entorhinal cortex (EC), recent findings show that EC LII neurons expressing wolfram syndrome 1 (Wfs1+) project to CA1, and stellate neurons in EC LII expressing reelin (Reelin+) project to dentate gyrus and CA3 (please see Kitamura et al., 2014, 2015). **B** A circuit diagram of the specific cell types participating in entorhinal- hippocampal circuitry (please see Xu et al. 2016).

Though human AD has long been characterized by amyloid (Aβ) plaques, and neurofibrillary tangles (NFTs) composed of misfolded, hyperphosphorylated tau proteins [13, 14], the causal link between neuropathological features and neural circuit/cognitive dysfunction in AD remains unclear (reviewed in Herrup, 2021). Neural circuit dysfunction is increasingly being acknowledged as contributing to the neuropathological features of AD [15]. Early dysregulation of the entorhinal-hippocampal (EC-HPC) system circuitry, which is well-known for memory function starting with clinical studies of the patient HM [16, 17], is strongly correlated with AD progression. This dysregulation follows an inverse U-shaped trend in AD progression: there is a very early stage of neural circuit hyperactivity followed by late stage hypoactivity [18]. Physiologically, these damaging patterns of dysregulated neural activity spread throughout the EC-HPC circuitry, contributing to neuropathology [19]. This cascade of physio-pathological events culminates in the cumulative damage of those circuits at later stages [9], and results in the loss of memory that is associated with AD dementia [9, 20–23]. Significant progress has been made toward characterizing the EC-HPC system circuitry, particularly in animal models (Fig. 1). It will be important to learn about what aspects of the EC-HPC circuit are susceptible to AD.

## Characterizing AD with single-cell, spatial, and circuit omics

### Technologies applied to AD

Due to their invasive nature, there are clear limitations of applying omics technologies for studying the brains of AD patients. Yet, using postmortem brain tissue there has been considerable progress in characterizing the identities of the cell types affected by AD across different stages of the disease [24, 25]. Single-nuclei RNA-seq has been used to generate transcriptomic data (42,528 nuclei) from the entorhinal cortices (EC) of 10 patients at different Braak stages of AD [26]. Interestingly, in one study a subset of excitatory neurons, the RORB (RAR-related orphan receptor B)-expressing neurons, are most susceptible to AD early in disease progression. Reactive astrocytes are also more prevalent in EC at relatively early AD stages, and they express fewer markers of neural homeostasis and synaptic maintenance. These results demonstrate the co-involvement of neural and glial cell processes early in AD, and highlight the use of omics to characterize the identities of the cells affected by AD as well as their complex interactions, as astrocyte dysregulation may mediate neural circuit hyperactivity in AD [27, 28]. Several studies to date have used single nucleus transcriptomic technologies to characterize cells in the EC-HPC system in both human AD patients and animal models of AD (see Table 1).

**Table 1:** Single cell/Nucleus transcriptomics applied to the EC-HPC system in both human AD patients and animal models of AD.

Species	Method	Region
*Homo Sapiens*	sn-RNA-seq	hippocampus
	*N* = 13	"A Single-Cell Transcriptome Atlas of Glia Diversity in the Human Hippocampus across the Lifespan and in Alzheimer’s Disease."
		Su et al., 2022, *(unpublished)*
*Homo Sapiens*	sn-RNA-seq	hippocampus
	*N* = 6	"Molecular landscapes of human hippocampal immature neurons across lifespan."
		Zhou et al., 2022, *Nature*; PMID: 35794479
*Homo Sapiens*	sn-RNA-seq	hippocampus and prefrontal cortex
	*N* = 25	"A human brain vascular atlas reveals diverse mediators of Alzheimer’s risk."
		Yang et al., 2022, *Nature*; PMID: 35165441
*Homo Sapiens*	sn-RNA-seq	entorhinal cortex
	*N* = 24	"Diverse human astrocyte and microglial transcriptional responses to Alzheimer’s pathology."
		Smith et al., 2022, *Acta Neuropathol*; PMID: 34767070
*Homo Sapiens*	sn-RNA-seq	entorhinal cortex and superior frontal gyrus
	*N* = 20	"Molecular characterization of selectively vulnerable neurons in Alzheimer’s disease."
		Leng et al., 2021, *Nat Neurosci*; PMID: 33432193
Homo *Sapiens*	sn-RNA-seq	entorhinal cortex
	*N* = 8	"A single-cell atlas of entorhinal cortex from individuals with Alzheimer’s disease reveals cell-type-specific gene expression regulation."
		Grubman et al., 2019, *Nat Neurosci*; PMID: 31768052
*Homo Sapiens*	spatial transcriptomics	superior frontal gyrus
	*N* = 3	"Spatial transcriptomics and in situ sequencing to study Alzheimer’s disease."
		Chen et al., 2020, *Cell*; PMID: 32702314
*Mus Musculus*	sc-RNA-seq	hippocampus
	*N* = 6	"Desaturase inhibition reverses immune, synaptic and cognitive impairments in an Alzheimer’s disease mouse model."
		Hamilton et al., 2022, *Nat Comm*; PMID: 35443751
*Mus Musculus*	sc-RNA-seq	hippocampus
	*N* = 1480	"AD-linked R47H-TREM2 mutation induces disease-enhancing microglial states via AKT hyperactivation."
		Sayed et al., 2021, *Sci Transl Med*; PMID: 34851693
*Mus Musculus*	sc-RNA-seq	hippocampus
	*N* = 12	"TREM2-independent oligodendrocyte, astrocyte, and T cell responses to tau and amyloid pathology in mouse models of Alzheimer disease."
		Lee et al., 2020, *Cell Rep*; PMID: 34965428
*Mus Musculus*	sn-RNA-seq	hippocampus
	*N* = 6	"Computational Repurposing of Bumetanide for Preventing or Treating Alzheimer’s Disease."
		Taubes et a., 2021, (*unpublished*)
*Mus Musculus*	sc-RNA-seq	hippocampus
	*N* = 4	"Hippocampal glucose uptake as a surrogate of metabolic change of microglia in Alzheimer’s disease."
		Choi et al., 2020, *J Neuroinflammation*; PMID: 34465358
*Mus Musculus*	sn-RNA-seq	hippocampus
	*N* = 8	"GSAP regulates lipid homeostasis and mitochondrial function associated with Alzheimer’s disease."
		Xu and Wang, 2021, *J Exp Med*; PMID: 34156424
*Mus Musculus*	sn-RNA-seq	hippocampus
	*N* = 34	"Neuronal ApoE Upregulates MHC-I Expression to Drive Selective Neurodegeneration in Alzheimer’s Disease."
		Zalocusky et al., 2021, *Nat Neurosci*; PMID: 33958804
*Mus Musculus*	sc-RNA-seq	hippocampus
	*N* = 6	"Selective removal of astrocytic APOE4 strongly protects against tau-mediated neurodegeneration and decreases synaptic phagocytosis by microglia."
		Wang et al., 2021, *Neuron*; PMID: 33831349
*Mus Musculus*	sc-RNA-seq	hippocampus
	*N* = 12	"TREM2-independent oligodendrocyte, astrocyte, and T cell responses to tau and amyloid pathology in mouse models of Alzheimer disease."
		Lee at al., 2021, *Cell Rep*; PMID: 34965428
*Mus Musculus*	sc-RNA-seq	hippocampus
	*N* = 6	"Overexpressing low-density lipoprotein receptor reduces tau-associated neurodegeneration via apoE-dependent and independent mechanisms."
		Shi et al., 2021, (*unpublished*)
*Mus Musculus*	sn-RNA-seq	hippocampus
	*N* = 11	"Single-nucleus RNA sequencing reveals transcriptional changes of hippocampal neurons in APP23 mouse model of Alzheimer’s disease."
		Zhong et al., 2020, *Biosci Biotechnol Biochem*; PMID: 31928331
*Mus Musculus*	sn-RNA-seq	hippocampus
	*N* = 8	"Disease-associated astrocytes in Alzheimer’s disease and aging."
		Habib et al., 2020, *Nat Neurosci*; PMID: 32341542
*Mus Musculus*	sc-RNA-seq	hippocampus
	*N* = 2208	"Temporal Tracking of Microglia Activation in Neurodegeneration at Single-Cell Resolution."
		Mathys et al., 2017, *Cell Rep*; PMID: 29020624
*Mus Musculus*	spatial transcriptomics	hippocampus
	*N* = 15	"Spatial transcriptomics shows moxibustion promotes hippocampus astrocyte and neuron interaction."
		Zhang et al., 2022, *Life Sci*; PMID: 36220370
*Mus Musculus*	spatial transcriptomics	hippocampus
	*N* = 4	"Neurons burdened by DNA double-strand breaks incite microglia activation through antiviral-like signaling in neurodegeneration."
		Welch et al., 2022, *Sci Adv*; PMID: 36170369
*Mus Musculus*	spatial transcriptomics	hippocampus
	*N* = 4	"Cell type-specific inference of differential expression in spatial transcriptomics."
		Cable et al., 2022, *Nat Methods*; PMID: 36050488
*Mus Musculus*	spatial transcriptomics	hippocampus and olfactory bulb
	*N* = 6	"Spatial Transcriptomics Reveals Genes Associated with Dysregulated Mitochondrial Functions and Stress Signaling in Alzheimer Disease."
		Navarro et al., 2020, *iScience*; PMCID: PMC7522123
*Mus Musculus*	spatial transcriptomics	hippocampus
	*N* = 8	"Spatial transcriptomics and in situ sequencing to study Alzheimer’s disease."
		Chen et al., 2020, *Cell*; PMID: 32702314

To study the anatomical *locations* of cells in parallel with their transcriptomes, “spatial transcriptomics” has emerged as a powerful tool. Spatial transcriptomics is performed on brain tissue sections in situ coupled with imaging and sequencing/hybridization to visualize single cell resolution RNA expression [29, 30]. A recent study applied spatial transcriptomics to the hippocampi of an AD mouse model as well as human AD patients *post-mortem* [31]. This comparative study finds that myelination-related processes of oligodendrocytes are altered early in AD progression. Consistent with other studies, signs of inflammation, stress, and complement signaling are also observed, but later in AD progression and are more general spatially and in terms of cell type-specificity. These results show that early stages of AD are associated with cell type-specific and spatially-resolved transcriptomic processes. Several other studies have used spatial transcriptomics to study the EC-HPC system in the context of AD (Table 1). However, these single cell technologies alone do not allow researchers to characterize the wiring logic of the cell type-specific circuits affected by AD.

**Fig. 2:**
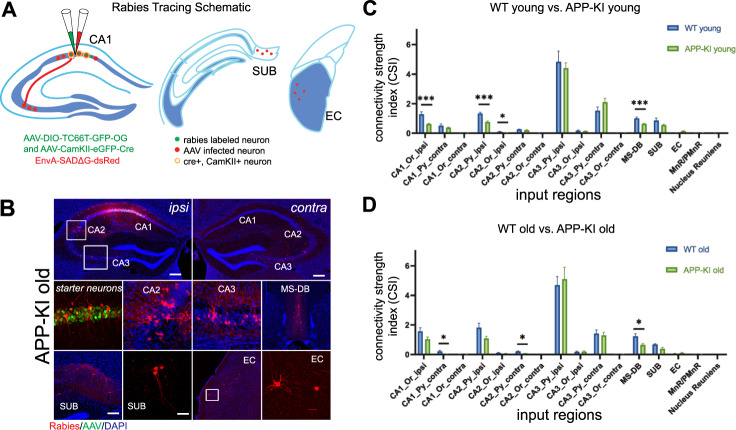
Viral tracing of the entorhinal-hippocampal system in an AD mouse model. **A** Schematic of cell type-specific retrograde monosynaptic rabies tracing. To label inputs to excitatory CA1 neurons, AAV helper virus (AAV-DIO-TC66T-GFP-oG and AAV-CaMKII-EGFP-Cre), labeled green, are injected into hippocampal CA1, followed by injection of rabies virus (EnvA-SADG-DsRed), labeled red. The neurons labeled both green and red are starter neurons. The neurons labeled only red represent the presynaptic inputs to the starter neurons. **B** Representative images from APP-KI old mice. Rabies virus-infected neurons are labeled by DsRed, and AAV-infected neurons are in green. Rabies virus mapped presynaptic inputs in the hippocampal regions CA1, CA2, anbd CA3 are shown in the sub-panels along with inputs from SUB and EC. **C**, **D** Quantitative analysis of CA1 CSI values across WT young and old mice and APP-KI young and old mice. The CSI is the ratio of the CA1 input neuron number in a subregion to the total starter neuron number in a brain (please see Ye et al. 2022).

To characterize the wiring logic of the cell type-specific *circuitry* affected by AD, methodologies must be used that retain information about the circuit properties of those cells (Table 2). For trans-neuronal monosynaptic tracing of cell type-specific circuits, herpes simplex type-1 virus (HSV-1 H129) and rabies virus are frequently used for the anterograde and retrograde directions, respectively [32]. The yellow fever vaccine (YFV-17D) is also effective for anterograde labeling [33]. For semi-quantitative analysis of tracing experiments, a commonly used metric is the connectivity strength index (CSI), defined as the ratio of the number of presynaptic neurons in each brain region versus the number of starter neurons in a brain region of interest [34, 35]. Recently, the monosynaptic rabies tracing method has been applied by Ye et al. 2022 to the EC-HPC system of the single APP-knockin AD model to determine how local and global circuit connectivity to hippocampal CA1 excitatory neurons is altered with AD progression [36]. Broadly, inputs to hippocampal region CA1 decrease with age and with AD progression, including inputs from CA1 itself and from CA2. Proportional inputs to CA1 from CA3 increase with age and AD progression, and this notable result suggests potential compensatory mechanisms associated with functional circuit remodeling and reorganization in response to disease progression [36]. Thus, genetically targeted neural circuit tracing can be used to gain new insight into the cell type-specific circuit connectivity and wiring logic of the EC-HPC system in AD models (Fig. 2).

**Table 2:** Genetically-targetted neural circuit tracing of the EC-HPC system in AD models.

Species	Method	Region
*Mus Musculus*	Rabies Tracing	hippocampus (CA1)
	*N* = 7–10	"Hippocampal neural circuit connectivity alterations in an Alzheimer’s disease mouse model revealed by monosynaptic rabies virus tracing."
		Ye et. al., 2022, *Neurobiol Dis*; PMID: 35843448
*Mus Musculus*	Rabies Tracing	hippocampus (CA1)
	*N* = 5	A novel mechanism of memory loss in Alzheimer’s disease mice via the degeneration of entorhinal–CA1 synapses.
		Yang et al., 2018, *Mol Psychiatry*; PMID: 27671476
*Mus Musculus*	Rabies Tracing	hippocampus (CA1)
	*N* = 3	"Dysfunction of Somatostatin-Positive Interneurons Associated with Memory Deficits in an Alzheimer’s Disease Model."
		Schmid et al., 2016, *Neuron;* PMID: 27641495
*Mus Musculus*	Rabies Tracing	hippocampus (CA1)
	*N* = 4	Early synaptic deficits in the APP/PS1 mouse model of Alzheimer’s disease involve neuronal adenosine A2A receptors.
		da Silva et al., 2016, *Nat Communs*; PMID: 27312972
*Mus Musculus*	Rabies Tracing	hippocampus (CA1)
		Assessment of a novel tau propagation pathway from layer II medial entorhinal cortical neurons to CA1 pyramidal neurons as an early BRAAK stage mouse model.
		Delpech et al., 2020, *Alzheimer’s & Dementia*
*Mus Musculus*	Rabies Tracing	hippocampus (CA3)
		Hippocampal Mossy Fibers Synapses in CA3 Pyramidal Cells Are Altered at an Early Stage in a Mouse Model of Alzheimer’s Disease.
		da Silva et al., 2019, *Neurobiol of Dis*; PMID: 30886015
*Mus Musculus*	Rabies Tracing	hippocampus (dentate gyrus)
		Activity-dependent reconnection of adult-born dentate granule cells in a mouse model of frontotemporal dementia.
		Moreno-Jimenez et al.,, 2020, *Alzheimer’s & Dementia*
*Mus Musculus*	Rabies Tracing	hippocampus (dentate gyrus)
	*N* = 5	Impairments of spatial memory in an Alzheimer’s disease model via degeneration of hippocampal cholinergic synapses.
		Zhu et al., 2017, *Nat Commun*; PMID: 29162816
*Mus Musculus*	Rabies Tracing	entorhnal cortex (EC)
		Wolframin-1–expressing neurons in the entorhinal cortex propagate tau to CA1 neurons and impair hippocampal memory in mice.
		Delpech et al., 2021, *Scie Transll Med*; PMID: 34524859
*Mus Musculus*	Rabies Tracing	entorhinal cortex (EC)
		Mapping entorhinal cortex circuitry in mouse models of Alzheimer’s Disease.
		Macchia and Beier, 2022, *Alzheimer’s & Dementia*
*Mus Musculus*	Rabies Tracing	prefrontal cortex (PFC)
	*N* = 3	"Acetylcholine deficiency disrupts extratelencephalic projection neurons in the prefrontal cortex in a mouse model of Alzheimer’s disease."
		Sun et al., 2012, *Nat Communs;* PMID: 35194025
*Mus Musculus*	Rabies Tracing (antero)	hippocampus (dentate gyrus)
	*N* = 4	"An anterograde rabies virus vector for high-resolution large-scale reconstruction of 3D neuron morphology."
		Haberl et al., 2015, *Brain Struct and Funct*; PMID: 24723034
*Mus Musculus*	HSV1-H129	hippocampus (CA1)
	*N* = 3	"Posterior basolateral amygdala to ventral hippocampal CA1 drives approach behavior to exert an anxiolytic effect."
		Pi et al., 2020, *Nat Commun*; PMID: 31924799
*Mus Musculus*	HSV1-H129	prefrontal cortex (PFC)
	*N* = 3	"A novel H129-based anterograde monosynaptic tracer exhibits features of strong labeling intensity, high tracing efficiency, and reduced retrograde labeling."
		Yang et al., 2022, *Molr Neurodegener*; PMID: 35012591

Characterization of the identities, locations and circuitry of the EC-HPC system cells affected earliest by AD will provide the basis for functional studies. In the early stages of AD, initial responses to disease-mediated dysregulation in the EC-HPC system appears to be specific to cell types with defined neuroanatomy and wiring, although much more research in this direction is needed. Whereas in later stages of AD the affected cells appear to be more generic. These approaches will likely need to be complemented with other neuroanatomical imaging modalities [37, 38], which will reveal cell type-specific circuit structural changes early in AD progression.

### Possible future technological developments

In recent years there has been rapid progress in the development of single-cell omics to characterize the brain [39–42]. In 2021 the BRAIN Initiative Cell Census Network (BICCN) published a collection of 17 papers. The flagship paper in this collection detailed with unprecedented comprehensiveness a cell census, atlas, and wiring diagram in cortex for mice, marmosets and humans [43]. State-of-the-art sn-RNA-seq technologies were used to generate transcriptomic data [44], in situ hybridization and sequencing was used to generate spatially resolved transcriptomic data [45], and anterograde and retrograde labeling was used to generate wiring logic data for specific projection neurons [46, 47]. When applied in concert, these approaches provided the identities, locations, and wiring diagrams for a large number of specific neuronal cell types [48].

The combination of transcriptomic approaches, including spatial transcriptomic, with circuit tracing approaches is a promising synthesis that will be applied to the study of the EC-HPC circuitry in the context of AD. Extensive work has already been done using sparse labeling with barcoded viruses, such that the projection sites of neurons in a particular brain region can be determined in a high-throughput manner [49–51]. Building on this approach, recently developed spatial transcriptomic methods with cell type-specific resolution has been employed to mark the neuroanatomical position of each individual projection neuron along with its projection target regions [52, 53]. This general approach can be extended by integrating barcoded trans-neuronal viral tracing with spatial transcriptomics. This will reveal cell type-specificity and the neuroanatomical position of individual projection and target neurons. These technologies will provide rich datasets detailing the identities and characteristics of individual neuron connections first affected by AD in EC-HPC circuits.

## Functionally manipulating cell type-specific circuits in the AD brain

### Technologies applied to AD

How EC-HPC system dysregulation contributes to AD can be worked out by functionally manipulating cell type-specific circuitry. In the EC-HPC system, *hippocampal region CA1* hyperactivity is present early in clinical AD progression. To determine how CA1 circuit dysregulation might mechanistically contribute to hippocampal AD pathology, one study used a chemogenetic approach to manipulate CA1 circuit activity in mouse models of AD [54]. For the “Designer Receptors Exclusively Activated by Designer Drugs” (DREADDS) chemogenetic approach, modified receptors are expressed by neurons that are not activated by endogenous ligands but instead are exclusively activated by exogenous synthetic ligands, aka “Designer Drugs”. A DREADDs version of the modified M3 muscarinic receptor hM3Dq is activated by the artificial ligand clozapine N-oxide (CNO, a metabolite of clozapine [55]). CNO binding to hM3Dq results in membrane depolarization and neuron activation following the activation of phospholipase C (PLC) cascade signaling. A DREADDs version of the M4 muscarinic acetylcholine receptor, hM4Di, when used with CNO, results in membrane hyperpolarization (deactivates neurons) through a decrease in cyclic adenosine monophosphate (cAMP) signaling [56]. As both AD models that were used in this study have CA1 hyperactivity early in disease progression, inhibitory DREADDs (hM4Di) was used to counteract CA1 hyperactivity. This DREADDs-mediated inhibition of the CA1 neural circuitry reduces this hyperactivity as is expected. Very importantly, this intervention of lowering neural activity using DREADDs also attenuates AD-like pathology and Aβ deposition in regions containing axons or dendrites of DREADD-expressing neurons [54]. Reciprocally, DREADDs-mediated excitation of neural circuitry in wild-type (WT) controls, using excitatory DREADDs (hM3Dq) increases AD-like pathology. This study highlights how neural activity contributes to AD neuropathology and is supported by clinical and mouse model data showing that dysregulation of hippocampal circuit activity is a very early feature of AD and may initiate other pathology [9].

Entorhinal cortex (EC) hyperactivity is also present early in clinical AD progression and likely temporally follows CA1 circuit dysregulation (Braak Stages of AD) [57]. Aberrant neural activity patterns may spread from CA1 to EC since CA1 projects to the subiculum (SUB) [58, 59], which then projects to EC. CA1-EC communication is very strong as CA1 “place cells”, which are active when an animal enters a spatially localized region (“place”) in its environment, and EC “grid cells”, which fire at regular intervals (“grids”) to allow animals to understand their position in space, both contribute to specific memory processes relevant to AD [60]. Once dysregulated neural activity spreads from CA1 to EC, grid cells exhibit altered tuning properties [61–64]. In functional studies designed to measure the effect of EC dysregulation on AD pathology, DREADDs were used to experimentally manipulate EC neural activity. Localized inhibition of the EC neural circuitry reduces AD pathology in EC in a fashion that is similar to the effects described following manipulation of activity in CA1 above. Surprisingly, DREADDs-mediated manipulation in EC also reduces neuropathology in hippocampus [65]. Collectively, these results suggest that neural dysregulation in hippocampus influences the health of the EC, and vice versa. Functional dysregulation of the EC-HPC system is an important mechanistic feature of AD progression and reversing this attenuates the neuropathological features of the disease.

EC *hypoactivity* has been observed later in AD progression, coinciding with when memory loss is clinically observed. Mouse models of AD exhibit this pattern as well, as EC hypoactivity and dysregulated grid cell tuning appears late in AD progression when animal memory function is significantly impaired [66, 67]. This pattern of neural hypoactivity may then spread back to CA1 resulting in significant deficits in hippocampal function [68]. EC “island cells” are a type of grid cell projecting to CA1 that express wolframin-1 (Wfs1 + ). Dysregulated EC island cells may be responsible for propagating hypoactivity throughout the EC-HPC system [69–72]. To test this, optogenetic manipulation of Wfs1+ EC cells was used. For optogenetic manipulation of neurons, modified opsins [73], which are light-activated via a fiber optic cable [74], are expressed by the neurons of interest. Light activated ion transfer across the neural membrane of these neurons activates or inhibits cell type-specific circuitry. Blue light-activated cationic charge carrier Channelrhodopsin-2 (ChR2) is used to activate neurons by membrane depolarization. Halorhodopsin and archaerhodopsin are yellow and green light-sensitive anion carriers, respectively; they inhibit neurons by light-activated hyperpolarization [75]. In control animals optogenetic excitation (ChR2) of Wfs1+ cells in EC results in robust increases in CA1 neuronal activity. In contrast in a mouse AD model, excitation of Wfs1+ neuron axons results in relatively lower levels of CA1 excitation as shown by multielectrode array recordings. These results support the idea that the EC→CA1 circuitry is weakened in AD and may contribute to functional loss [76]. DREADDs-mediated activation of CA1 in the AD mouse model also results in relatively less CA1 activity compared to control animals as measured by cFOS staining. Together, these results support the idea that EC hypoactivity in the late stages of AD contributes to CA1 hypoactivity [70].

The SUB is situated anatomically between CA1 and the EC. However, the role of the SUB in AD disease progression in the context of CA1 and EC circuitry is not yet known. Anatomically, the CA1 to SUB forward projection (CA1→SUB) is well-established. There are direct connections from CA1 to SUB, as cells that express teneurin-3 (Ten3 + ) or latrophilin-2 (Lphn2 + ) project to the SUB [58, 59]. There is also an important SUB to CA1 back-projection pathway (CA1←SUB) that has only recently been discovered [35, 77–79]. The function of this SUB to CA1 back-projection was determined using long-term trans-synaptic expression of optogenetic and chemogenic constructs. Retro-adeno-associated virus type 2 (rAAV2-retro) or Canine adenovirus type 2 (CAV-2) – which both spread retrogradely – are useful for this purpose due to their lower toxicity relative to other viruses [32, 80–82]. rAAV2-retro virus expressing Cre recombinase was injected into the CA1 region of transgenic mice to express a Cre-dependent optogenetic construct to activate CA1-projecting excitatory SUB neurons [79]. Injection of CAV2 virus expressing Cre recombinase into the CA1 region was also performed in combination with injection of AAV expressing a Cre-dependent chemogenetic construct in SUB to specifically modulate the activity of CA1-projecting excitatory SUB neurons [79]. It was determined that the SUB to CA1 back-projection pathway (CA1←SUB) facilitates memory processing in CA1 [79]. The SUB to CA1 back-projection pathway has not previously been studied in the context of AD. Given the SUB’s anatomical location between CA1 and EC, it is very well-positioned to be a critical bidirectional mediator of AD disease progression between these brain regions.

The extended EC-HPC system is a very interesting piece of brain circuitry that is clearly implicated in AD. Increasing our understanding of how dysregulation of the EC-HPC circuitry leads to AD has made considerable progress in recent years, though these studies are still in a relatively nascent stage. One of the goals of this Review article is to excite AD researchers to use available technologies and concepts to probe the EC-HPC circuit basis of AD, as it has become clear that AD physiology and pathology are causally interconnected.

### Possible future technological developments

The previous section describes novel discovery-focused technologies. It is important to verify putative characterizations with functional studies of cell type-specific circuits [12, 83]. To do this, cell type-specific circuits relevant to AD must be targetable in some way [26]. This requires genetic information about cell types and specific circuits that can be used to create a “genetic toolbox” for cell type-specific targeting of the EC-HPC system [84]. Promoter/enhancer-based mouse driver lines and viral tools for targeting cell type-specific circuits have expanded rapidly over the past several years [85–92]. When coupled with intersectional genetic approaches [91, 93], these mouse lines and viruses can be used to target even more refined cell type-specific circuitry for functional modulation.

Once a genetic approach is identified that narrows cell type-specific circuit targeting, a functional assay that permits modulation of activity in the circuit of interest is used to test physiological and behavioral function [94, 95]. Optogenetics is the most widely-used technique for modulating neural activity with temporal precision. One shortcoming of optogenetic approaches is that they can be invasive as short wavelength light-activated optogenetic proteins impose limits of tissue penetration. Therefore, optic fibers are inserted into the brain to deliver light to the region of interest. The use of red light-sensitive opsins such as Chrimson can overcome the issue of invasively inserting hardware into the brain as red light may activate associated opsins down to brain depths between 1 and 2 cm, potentially from outside the skull [96–99]. This approach can be adopted to non-invasively modulate EC-HPC circuits implicated in AD progression.

## Understanding why specific circuits are implicated in AD

### Technologies applied to AD

The EC-HPC system is a potential point of convergence for many diverse AD-associated issues [100]. Thus, the integration of the EC-HPC system’s circuitry with the memory-related behavioral needs of the animal may further contribute to a pathological feedback cycle of dysregulated circuit activity. Memory is encoded in the hippocampus by “engram cells”, and depending on the memory-related needs of the animal, memories can be retrieved by these engram cells at a later time [101]. Surprisingly, the *encoding* of memories by engram cells is not disrupted in animal models of AD during behavior. This can be shown by the direct optogenetic activation of hippocampal engram cells, which results in memory retrieval and behavior in otherwise amnesic mice. Rather, it is impairments in memory *retrieval* that results in reduced memory performance in AD mice even before plaque deposition [102–105]. A potential interpretation of this result is that the AD brain may adaptively shunt neural activity away from the EC-HPC circuitry as the memory-related behavioral needs of the animal are overwhelmed.

**Fig. 3:**
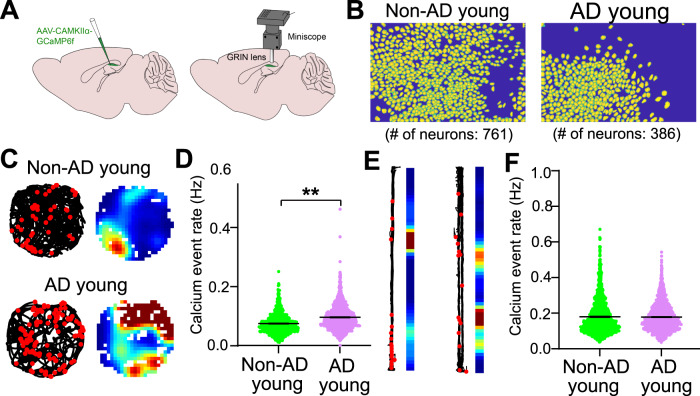
CA1 neuron ensembles in AD models have increased activity in complicated behavioral environments compared to control mice. **A** Schematic of in vivo neural calcium imaging of hippocampal CA1 excitatory neurons in 3xTg-AD and Non-Tg (non-AD) mice (3–6 m.o.) with miniscopes in freely behaving animals. **B** Examples of neuron footprints from CNMF-E extraction for data processing for young non-AD and young AD mice. **C** (left) Travel trajectory in a circular arena plotted by the black line, and red dots are where the calcium events are higher than the threshold. (right) Spatial rate maps of calcium events during exploration of the circular arena. **D** Violin plots of calcium event rates of all neurons from non-AD controls and 3x-Tg AD mice during exploration of the circular and square arenas. **E**, **F** Travel trajectories, spatial rate maps, and violin plots of calcium rates for non-AD and 3x-Tg AD mice exploring a linear track. **D**, **F** CA1 calcium event rates are significantly increased in the AD model during exploration of circular and square arenas, but not during exploration of the track (please see Lin et. al. 2022).

A recent study provides more insight into why the EC-HPC circuitry is implicated in AD progression in terms of the memory-related behavioral needs of the animal. Lin et al. (2022) recently investigated hippocampal region CA1 circuit hyperactivity in a freely behaving AD mouse model using miniscope-based GCaMP calcium imaging [106]. The study used the 3xTg-AD mouse line which contains mutations that lead to both Aβ and tau pathology [107–110]. Intriguingly, CA1 neurons are found to be hyperactive but only during specific behaviors [106]. CA1 circuit hyperactivity is observed in open field behaviors, but not in linear track behaviors. This suggests that simple behaviors that do not require higher cognitive load, and thus extensive computation in CA1, may not evoke deficits in hippocampal circuit operations in AD models (Fig. 3). This result reinforces the idea that the EC-HPC circuitry is inundated with neural activity early in AD, and that the increasing performance demands of memory-related behaviors exacerbate this effect. Importantly, clinical research shows that more difficult memory tasks recruit increased hippocampal activity, and that this increase in hippocampal activity is even more pronounced in AD patients than in healthy individuals [111–113].

**Fig. 4:**
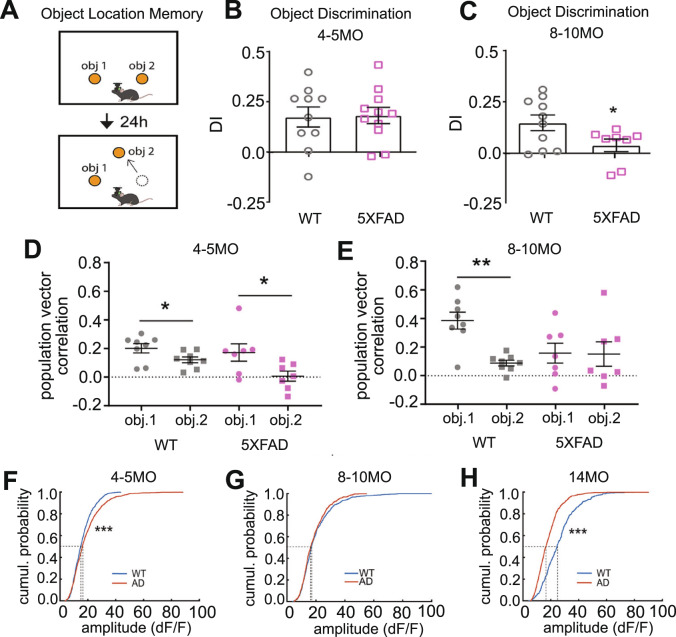
Longitudinal measurements of CA1 neuron calcium activity and memory behavior in an AD model reveals age-dependent relationship. **A** Schematic of the object location memory (OLM) test whereby mice are exposed to two identical objects in the training session and then are tested after 24 h with one object moved to a new location. Discrimination indexes (DIs) are calculated during the testing session. **B**, **C** 5xFAD mice do not show a decreased DI until 8–10 MO, and have normal DI at 4–5 MO. **D**, **E** Population vector correlations for each object at 4–5 or 8–10 months of age. WT and AD mice show significantly higher correlation for the unmoved object (obj1) than the moved object (obj2) at 4–5MO, whereas, while 5xFAD mice show similarly low correlation for both objects at 8-10MO. **F**–**H** Calcium event amplitudes of CA1 neurons during the overall session of exploring a circular arena at different testing ages. CA1 neurons of 5xFAD mice show a gradual change in overall amplitude compared to WT from 4-5 to 14 months of age (please see Zhang et. al. 2023).

**Fig. 5:**
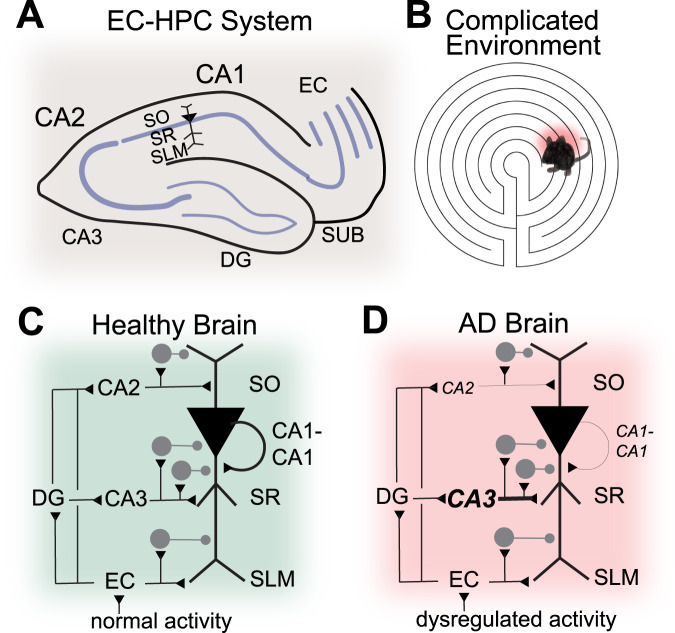
Entorhinal-hippocampal system impacted by AD. **A** A cartoon representation of the entorhinal-hippocampal (EC-HPC) neuroanatomy in mouse. **B** A cartoon depiction of increased HPC activity in AD animals in complicated behavioral environments (please see Lin et. al., 2022). **C** A wiring diagram of the EC-HPC circuitry in normal healthy animals. **D** A wiring diagram of the EC-HPC circuitry in AD animals. Note the decreased connectivity of CA1 and CA2 to CA1 neurons, as well as the proportionally increased connectivity of CA3 to CA1 neurons in AD animals (please see Ye et. al. 2022). It is not yet clear if these structural changes are adaptive or a result of neurodegeneration. As the neural circuitry activity of the AD brain becomes dysregulated, memory intensive behaviors overwhelm the EC-HPC system revealing its deficits.

Another very recent study provides more insight into why the EC-HPC circuitry is implicated in AD progression in terms of the memory-related behavioral needs of the animal. Zhang et al. (2023) recently investigated hippocampal region CA1 circuit activity and memory-related behavior longitudinally in an AD mouse model using miniscope-based GCaMP calcium imaging [114]. This study used the 5xFAD mouse line which carries 3 mutations in human amyloid precursor protein (APP) and 2 mutations in presenilin 1 (PSEN1), inducing early and rapid amyloid aggregation before the emergence of memory deficits [115–117]. Intriguingly, CA1 neuron activity profiles from 5xFAD animals are significantly different from WT at a young age (4–5MO) where AD mice do not yet display behavioral disturbances determined using the object location memory (OLM) test—but they do exhibit measurable circuit defects. Yet, at an older age (8–10MO) when AD mice do have robust reductions in OLM performance, aspects of CA1 neuron activity profiles are not significantly different from WTs. At an even older age (14MO), when OLM performance in the AD mice is even worse than at 8–10MO, 5xFAD CA1 neuron activity profiles are again significantly different from WTs, but in the opposite direction seen at 4–5MO. This longitudinal relationship between memory-related behavior changes and the activity profiles of CA1 hippocampal neurons suggests that the EC-HPC system in AD mice may adaptively compensate to AD-related dysregulation, and further reflects the shift between circuit hyperexcitation early in the disease process followed by circuit hypo-excitation seen later in the disease process (Fig. 4). Importantly, clinical research has shown that patients with AD-related neuropathology and/or EC-HPC circuit activity changes adaptively compensate for these disturbances such that their memory performance appears normal until relatively later stages of disease progression [118] (Fig. 5).

### Possible future technological developments

The EC-HPC circuitry must be studied within the broader context of the brain and the animal’s behavior to understand why it is susceptible to AD. To do this, measurement and/or manipulation of EC-HPC circuit activity and/or memory-related behavior [51, 95, 119] can be performed to test theories about why EC-HPC circuits are functionally implicated in AD [120–122]. One new technology that will be very powerful for this purpose is “Comprehensive Readout of Activity and Cell Type Markers” (CRACK). CRACK combines population calcium imaging of circuit-specific neurons with subsequent multiplexed fluorescent in situ hybridization. Using CRACK, brain-wide projections of identified cells along with their behaviorally-defined tuning properties were recently integrated to build a model of specific circuit functions [123]. Thus, application of this approach to understanding AD models is feasible. Going forward, adaptations of the CRACK approach will reveal near-idealized linkages of the identities, locations, and wiring diagrams of cell type-specific circuits with longitudinal neural activity patterns and animal behavioral responses to well-controlled stimuli [124, 125]. Virtual reality (VR) stimuli can be used during calcium imaging of neural activity to study the function of hippocampal circuits during spatial navigation and memory-related behavior [126–128]. Together these methods can be applied to AD models to determine why the EC-HPC circuit is susceptible to AD, why dysregulation proceeds at different rates in different EC-HPC cell type-specific neurons circuits, and if any of these early changes in the EC-HPC circuitry are adaptive responses to AD-related processes throughout the brain.

## Potential entorhinal-hippocampal circuit-specific therapies for AD

The goal of AD research is to decipher the inner workings of the brain, and to leverage that knowledge to prevent and cure the disease [11]. By understanding why specific neural circuits are implicated in AD, it may be possible to develop therapies for AD that do not just treat “symptoms” but the disease itself. One therapeutic approach for doing this in the brain that is making rapid headway is gene therapy [129, 130]. Gene therapy is a medical approach for delivering genetic material to cells, typically using either virus [131, 132] or nanoparticles [133] with the goal of disease treatment. There are several approaches for gene therapy [129], including: introducing a new gene (gene delivery), modulating an existing gene’s activity (typically using oligonucleotides [134]), or the editing of a gene (CRISPR/Cas9 [135]). Gene therapy provides clinicians with a wide variety of potential approaches for modulating brain circuitry to treat AD.

For these therapies to reach the brain they must first successfully cross the blood-brain-barrier (BBB) [136]. Directed evolution of the AAV capsid protein has overcome this hurdle by generating new AAV serotypes (AAV.PHP.B [137]) that cross the BBB [138, 139]. As a result of this recent innovation and other factors, many AAV-mediated gene therapies for CNS disorders are being used in clinical trials [140] and several are FDA-approved [141], including an AAV-based gene therapy for RPE65 gene mutations responsible for retinal dystrophy. The Allen Institute and BioMarin have teamed up to generate enhancer AAVs that target cell type-specific circuitry for gene therapy in the human brain [91, 92, 142]. Similarly, nanoparticle-mediated gene therapy for CNS disorders is being developed [143], although only one therapy is FDA-approved [144]. Intranasal delivery may be a potential route of administration to allow efficient bypassing of the BBB for gene therapy-mediated treatment of AD using nanoparticles [145]. Recently, both AAV- and nanoparticle-mediated gene therapies have been used to express genes, alter gene expression, or effectively edit genes in the brain [146, 147].

Gene therapies directed to the EC-HPC system may be designed to treat AD patients with early symptoms. Such patients are likely to progress to full-blown AD dementia eventually without an effective intervention. As neuronal loss is a feature of AD at the later stages of the disease associated with significant cognitive and memory loss, using gene therapy to provide trophic support to remaining neurons in the EC-HPC circuitry is a reasonable approach. AAV-mediated gene therapy delivery of brain-derived neurotrophic factor (BDNF) is currently being tested for this purpose. BDNF promotes neuronal function in the EC-HPC system. In rodent and non-human primate animal models of AD, therapeutic delivery of the BDNF gene to the EC-HPC system reverses neuronal loss in those circuits and promotes the building of new synapses [130, 148–150]. Pre-clinical work has led to a Phase I clinical trial starting in 2022 that is testing the efficacy of AAV2-BDNF gene therapy (stereotaxically administered to the EC-HPC under MRI guidance, as safe methods for targeting this circuitry have not yet been developed) for treating patients with MCI or AD (see clinicaltrial.gov identifier NCT05040217). This study is estimated to be completed in 2027.

## Conclusions, unanswered questions and future directions

Experiments designed to develop a more complete understanding of why specific neural circuits are implicated in AD are relatively new. Most AD research over the past several decades has focused on AD’s neuropathological features and assume causative linkage with downstream neural circuit and behavioral dysfunction [7]. However, decades of AD research show that the idea that this disease has some singular genetic or molecular cause(s) that can be targeted for therapy in the clinic has been significantly complicated by evidence showing that AD etiology is highly heterogenous [151]. Except for a few well-known genetic marks restricted in some cases to single families that make up a very small percent of all AD cases [152], there are many disease-related risk factors that are associated with AD severity. Ageing, sleep, exercise, sex hormones, immune reactivity, metabolism, stress, neurogenesis, viral infection, cell senescence, diet, diabetes, obesity, smoking, alcohol, and many others, have been shown to be important in AD. Diverse factors contribute to AD and determining the relative contribution of these factors that are relevant for a patient will be difficult.

The prospect of having to decipher why each of these diverse processes acts in specific brain circuit to affect AD pathophysiology is even more daunting. Like the search for a genetic/molecular cause of AD, the search for the brain loci of AD has been difficult. While early vulnerable AD brain regions have been identified, many major brain regions are implicated in AD, including the pons, locus ceruleus, tegmental areas, thalamus, hypothalamus, nucleus basalis of Meynert, habenula, putamen, caudate nucleus, ventricles, dura, cerebellum, amygdala, visual cortex, parietal cortex, temporal cortex, prefrontal cortex, cingulate cortex, and even optic nerve and spinal cord, and many others. AD researchers should embrace the diversity of factors (biological processes and brain regions) contributing to the disease and this will require the synthesis of findings that were previously thought to be unrelated.

Herein, we present the case that specific brain circuitry including the entorhinal-hippocampal system is implicated in AD and may be a point of convergence in the disease. It has been known for decades that the entorhinal-hippocampal system functions as a computational bottleneck in the brain, taking diverse inputs and using a few processes to generate diverse outputs [153, 154]. These architectural and functional features of the entorhinal-hippocampal system may render this circuit particularly susceptible to dysregulated neural activity arising from a variety of genetic and environmental causes in AD patients. Going forward the cell type-specific circuitry in the EC-HPC system first susceptible to AD should be identified, as well as any components which are adaptively compensating the disease, and studied toward the development of novel and targeted circuit therapies.
